# Rare mutations predisposing to familial adenomatous polyposis in Greek FAP patients

**DOI:** 10.1186/1471-2407-5-40

**Published:** 2005-04-15

**Authors:** Markos Mihalatos, Angela Apessos, Hans Dauwerse, Voula Velissariou, Aristidis Psychias, Alexander Koliopanos, Konstantinos Petropoulos, John K Triantafillidis, Ioannis Danielidis, George Fountzilas, Niki J Agnantis, Georgios Nasioulas

**Affiliations:** 1 Molecular Biology Research Center HYGEIA – «Antonis Papayiannis», Athens; 2 Center for Human and Clinical Genetics, Leiden University Medical Center, The Netherlands; 3 Cytogenetics Laboratory, Department of Genetics and Molecular Biology, Mitera Maternity and Surgical Center, Athens, Greece; 4 Hygeia Ofthalmos, Diagnostic and Therapeutic Center of Athens HYGEIA S.A., Athens, Greece; 5 Surgical Clinic, General State Hospital of Athens "G. Gennimatas", Athens, Greece; 6 Gastroenterology Department, Euromedica – Engephalos Diagnostic Center of Athens, Greece; 7 Gastroenterology Department, Peripheral General Hospital of Nikaia, Greece; 8 Gastroenterology Department Diagnostic and Therapeutic Center of Athens HYGEIA S.A., Athens Greece; 9 AHEPA Hospital, Aristotle University of Thessaloniki, Thessaloniki Greece; 10 Department of Pathology, Medical School, University of Ioannina, Ioannina, Greece

## Abstract

**Background:**

Familial Adenomatous Polyposis (FAP) is caused by germline mutations in the *APC *(Adenomatous Polyposis Coli) gene. The vast majority of *APC *mutations are point mutations or small insertions / deletions which lead to truncated protein products. Splicing mutations or gross genomic rearrangements are less common inactivating events of the *APC *gene.

**Methods:**

In the current study genomic DNA or RNA from ten unrelated FAP suspected patients was examined for germline mutations in the *APC *gene. Family history and phenotype were used in order to select the patients. Methods used for testing were dHPLC (denaturing High Performance Liquid Chromatography), sequencing, MLPA (Multiplex Ligation – dependent Probe Amplification), Karyotyping, FISH (Fluorescence In Situ Hybridization) and RT-PCR (Reverse Transcription – Polymerase Chain Reaction).

**Results:**

A 250 Kbp deletion in the *APC *gene starting from intron 5 and extending beyond exon 15 was identified in one patient. A substitution of the +5 conserved nucleotide at the splice donor site of intron 9 in the *APC *gene was shown to produce frameshift and inefficient exon skipping in a second patient. Four frameshift mutations (1577insT, 1973delAG, 3180delAAAA, 3212delA) and a nonsense mutation (C1690T) were identified in the rest of the patients.

**Conclusion:**

Screening for *APC *mutations in FAP patients should include testing for splicing defects and gross genomic alterations.

## Background

Germline mutations in the *APC *gene are the causative factor for the Familial Adenomatous Polyposis syndrome which follows an autosomal dominant inheritance pattern [1,2]. FAP patients develop multiple to thousands of colonic polyps before their second decade of life.

If polyps are left untreated they give rise to colorectal cancer in almost all cases. The syndrome has two forms, one referred to as classic FAP and one milder form referred to as attenuated FAP (AFAP) [3]. Most FAP patients develop certain extracolonic features [3,4]. Congenital Hypertrophy of the Retinal Pigment Epithelium (CHRPE) is present in the majority of FAP patients. Nearly 94% of *APC *germline mutations lead to a truncated protein product. Thirty three percent of *APC *mutations represent nonsense point mutations, 6% small insertions and 55% small deletions [5].

Recently, a number of studies have demonstrated the presence of less common *APC *mutations in FAP patients, such as large genomic rearrangements or splicing affecting mutations [6,7]. It is generally noted that large rearrangements are more likely to produce a classic FAP phenotype, while splicing mutations tend to result in attenuated FAP (AFAP) [8-13].

Large rearrangements account for up to one third of identified mutations in the *BRCA1 *and *hMLH1 *and *hMSH2 *genes which are responsible for other distinct or related types of cancer, respectively [14,15]. In *APC *the percentages are lower but according to recent reports may reach as much as 10% of identified mutations [16]. Recently, MLPA has been used in our laboratory, to successfully identify large deletions in the *hMSH2 *and *BRCA1 *genes [17,18].

Splice site mutations are a common inactivating event for several human genes estimated to account for about 15% of the mutations [19]. In the *ATM *and *NF1 *genes they represent about 50% of the identified mutations [20,21]. Most frequent targets of splice site mutations are the consensus GT and AG donor and acceptor dinucleotides respectively. However, several splice site mutations altering other exonic or intronic nucleotides neighboring the consensus sites, have been described [12,21,22]. Screening the corresponding genes' cDNA elucidates most splicing defects.

Nevertheless, these studies point out the need for more intensive mutation screening than coding region and splice junction sequencing. Finally, these studies address the issue that such uncommon mutations may have been underestimated due to the methods of mutation screening that have been generally used in the past decade.

In the present study, two different cases of FAP families carrying two uncommon *APC *mutations are presented: a splice site mutation on the fifth nucleotide of intron 9 and a large genomic deletion from one chromosome 5 encompassing around 250 Kbp, starting in intron 5 of the *APC *gene and extending beyond exon 15. We also further support our ongoing study on FAP presenting another four frameshift mutations of the *APC *gene identified in four unrelated patients.

Finally, we propose a simple algorithm for the *APC *genetic testing of FAP patients based on our experience after screening 96 individuals from 25 FAP families from our Greek cohort.

## Methods

### Patients

Patients were referred to our center and selected for genetic testing as previously described [4]. Ethical approval was obtained from the HYGEIA – Diagnostic and Therapeutic Center of Athens S.A. – hospital's advisory committee (ref.27931/23.10.2000) and all patients signed informed consent.

### DNA and RNA isolation

Genomic DNA and RNA were purified from peripheral blood leukocytes using standard extraction protocols, as already described previously [4].

### PCR amplification

All 16 exons (1–15, 10A) including splice junctions of APC were amplified by PCR (Polymerase Chain Reaction) as already described elsewhere [4,23]. The primers used were taken from other studies [1,4].

### dHPLC analysis

The WAVE DNA Fragment Analysis System (Transgenomic, Inc., USA) and associated WAVE-Maker™ software were used as previously described [23].

### Sequence analysis

Purification of the PCR products and automated cycle sequencing for both strands was performed on the ABI Prism^® ^310 Genetic Analyzer as previously described [4,23].

### RT-PCR

First strand synthesis was performed by denaturing approximately 500 – 1000 ng total RNA and random hexamers (5 μM final concentration) for 4 min at 70°C, followed by snap freezing on ice and addition of dNTPs (0.5 mM final concentration), 1 U/μl recombinant RNase inhibitor (Invitrogen, The Netherlands) and 200 U MMLV reverse transcriptase (Invitrogen, The Netherlands). The mixture was incubated at 37°C for 1 hour followed by denaturation of the enzymes at 95°C for 5 min. 4 μl of cDNA were used for subsequent PCR amplification. The primers used for PCR were described by Varesco et al (1994) [22] and amplify the cDNA region between exons 8 and 12 of the APC gene.

### Multiplex Ligation-dependent Probe Amplification (MLPA)

MLPA was carried out using the P043 APC kit (MRC-Holland, Netherlands) as instructed by the manufacturer. Fragment analysis was carried out on the ABI Prism^® ^310 Genetic Analyzer using TAMRA-500 provided by the manufacturer, as size standard. A peak pattern of 31 peaks ranging in size from 130 to 400 nt is obtained [24].

### Conventional cytogenetic analysis

Metaphase chromosome preparations were obtained from peripheral blood lymphocytes using standard techniques. Conventional cytogenetic analysis was carried out using high resolution GTG banding (1200 bands). Cells were analyzed, karyotyped and captured by computer imaging (Cytovision system, Image Analysis, Applied Imaging, Sunderland, UK).

### FISH analysis

FISH analysis was performed as already described by Dauwerse JG et al (1992) [25]. Cosmid probes cAPC1, cAPC2, cAPC3 for the APC gene and MCBCD, for the MCC gene on band 5q22.2 were simultaneously hybridized with the telomere PACs GS-240G13 (5qter) and GS-24H17 (5pter) for identification of chromosome 5 [26,27].

## Results

### Alternative splicing

In family A the proband was a young man at the age of 29 with a phenotype showing more than a hundred polyps throughout the colon, particularly in the rectum, sigmoid and descending colon. No polyps were found in the esophagus, duodenum and ileum, except for one polypoid adenomatous lesion in the stomach. The polyps were adenomatous, smaller than 5 millimeters and some tubular and serrated adenomas were also observed. All lesions showed low or medium grade dysplasia while some serrated adenomas were mildly hyperplastic. Funduscopic examination showed the absence of CHRPE.

**Figure 1:**
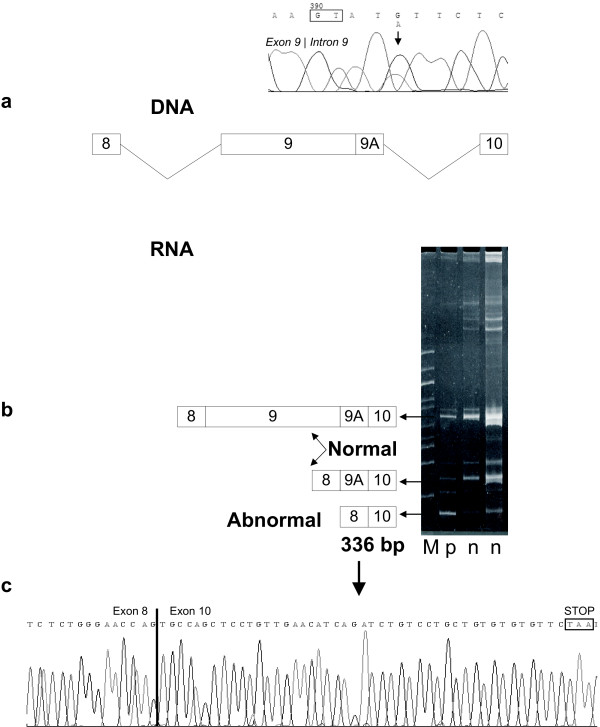
**A. **DNA sequence chromatogram at the splice junction between exon 9 and intron 9. Donor site is in a box and IVS9+5G>A mutation is indicated by an arrow. Genomic structure from the region including exons 8 and 10 with exons in boxes and introns printed as broken lines. **B. **The two normal splice variants 9 (exons 8-9-9A-10), 9A (exons 8-9A-10) and the abnormal variant missing exon 9 (exons 8–10). RT-PCR products on 6% PAGE are indicated by horizontal arrows. **M: **100 bp DNA Ladder (New England Biolabs), **p: **patient sample and **n: **normal samples. The 336 bp abnormal product is quite evident in the patient sample. **C. **Chromatogram showing sequence of the abnormal transcript. Vertical line in chromatogram showing the abnormal junction between exons 8 and 10 while the box indicates the STOP signal created by the frameshift.

dHPLC screening of the *APC *gene revealed an aberrant elution profile for the amplicon encompassing both exons 9 and 9A. Subsequent sequencing of that amplicon confirmed the presence of the IVS9+5G>A mutation (Fig. 1a). In order to examine the consequences of the G to A transition, RT-PCR was performed from total RNA extracted from peripheral blood lymphocytes of the proband. Total RNA from normal individuals and other FAP patients with identified mutations in other regions of the *APC *gene, were used as normal controls for this RT-PCR. RT-PCR products were separated by non denaturing 6% PAGE and visualized by EtBr staining (Fig. 1b). Two normal transcripts were amplified in all samples representing the two normal spliced forms of exon 9: 9 and 9A (Fig. 1b). A novel shorter transcript was only amplified from the proband's RNA (Fig. 1b). The corresponding band of 336 bp was purified and sequenced. Sequence analysis revealed an alternate transcript in which the whole of exon 9 was missing and exon 8 was directly joined to exon 10. This abnormal splicing results in a frameshift and creates a STOP signal at the 16^th ^codon of exon 10 (Fig. 1c).

The proband's phenotype was resembling AFAP with a late age of onset. The proband's siblings were asymptomatic but younger than 25 years old while the proband's parents were of normal phenotype. In order to exclude AFAP from these individuals, they were screened by dHPLC for the IVS9+5G>A mutation. All individuals were of normal genotype, indicating that the mutation is *de-novo*.

### Large genomic rearrangements

**Figure 2:**
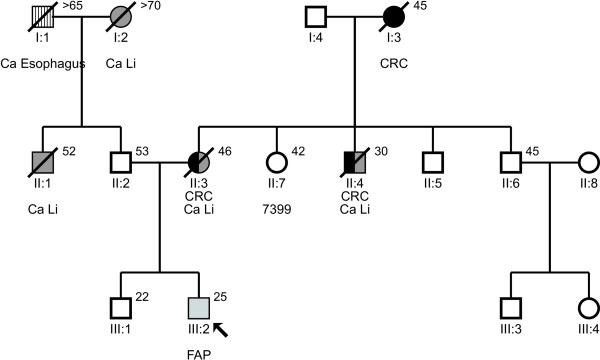
Family tree of the FAP family carrying a gross *APC *deletion. Though no sample is available for individuals I:3, II:3 and II:4, phenotype, cause and age of death indicate the presence and direct transmission of the deletion identified in the proband (III:2). (CRC: Colorectal Cancer, Ca Li: Liver cancer).

In family B the proband was a young man at the age of 25 with a phenotype of more than a hundred polyps throughout his colon. The patient's mother and maternal uncle died at the age of 46 and 30 from CRC and liver cancer – probably metastatic, respectively. The patient's maternal grandmother had died from CRC at the age of 45 (Fig. 2). Polyps were also present in the stomach, duodenum and ileum. The polyps were tubular adenomas, ranging in size from 5 to 15 millimeters and some with a diameter of more than 10 mm were pediculus. All lesions showed low or medium grade dysplasia. Funduscopic examination showed the presence of CHRPE for this patient.

**Figure 3:**
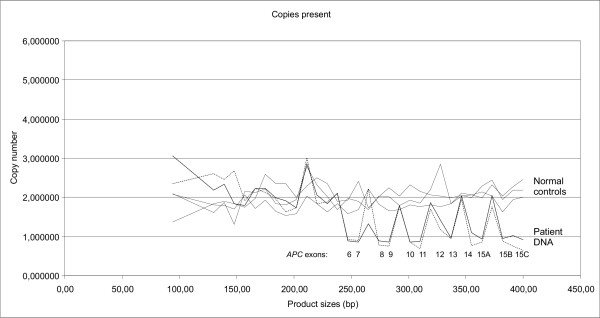
Results from MLPA experiment. Chart of normalized copy number of the MLPA probes target sequences, representing *APC *exons and control regions on other chromosomes, versus size of amplified products. Two different DNA samples from the same patient present only one copy per cell for exons 6 to 15 supporting the presence of a large *APC *deletion from one chromosome.

dHPLC and sequencing of the whole *APC *coding region, including splice junctions showed only the known G4479A polymorphism for which the patient was homozygous. Considering these results and the severity of the patient's phenotype, MLPA was performed to screen for genomic rearrangements which might had been undetected with the conventional techniques. MLPA analysis showed the deletion of exons 6 to 15 from one allele, in the patient's lymphocytes, from two different blood samples (Fig. 3). The proband's asymptomatic brother tested normal.

A large deletion, starting from intron 5 and ending downstream of the last *APC *exon, was revealed. In order to narrow down the 3' breakpoint, karyotype analysis was performed on the patient's peripheral blood lymphocytes. Karyotyping was negative excluding a deletion longer than 20 Mbp. Subsequent FISH analysis confirmed that a large portion of the *APC *gene was missing from one chromosome 5 (data not shown). Further FISH analysis showed that the 3' breakpoint of the deletion is lying in the *MCC *gene near its 3' end (data not shown). The 5' breakpoint lies somewhere in intron 5 which has a size of about 12 Kbp. These data indicate that the deletion encompasses about 250 Kbp.

### APC frameshift and nonsense mutations

Four frameshift mutations (1577insT, 1973delAG, 3180delAAAA and 3212delA) of the *APC *gene were identified in four unrelated FAP patients and the C1690T nonsense mutation was identified in two unrelated patients. To our knowledge 1577insT is a novel mutation.

In two patients no mutation was identified in the *APC *either screening for small insertions, deletions or point mutations or for large genomic rearrangements.

## Discussion

### Alternative splicing and exon skipping

In the current study the IVS9+5G>A mutation was identified and investigated. Our patient's phenotype is resembling more to AFAP than to FAP, with small polyps, particularly in the right colon. A novel transcript is produced in the carrier's peripheral blood lymphocytes not present in normal controls. Normal transcripts are also present in the patient's sample, but result in lower amplification when compared to the corresponding ones of the normal samples (Fig. 1a). The IVS9+5G>A mutation does not alter the splicing donor site but changes a very conserved nucleotide. Varesco *et al*, (1994) [22] have documented the transversion of G to T (IVS9+5G>T) at the same point to be pathogenic. The IVS9+5G>T mutation causes inefficient exon skipping, thus producing a milder phenotype leading to attenuated FAP. In both cases, the predicted truncated protein product of about 35 kDa is not large enough to stay stable and produce a dominant negative effect. The absence of CHRPE from this patient is in agreement with the findings from Olschwang *et al *(1993) [28] who concluded that patients with mutations before exon 9 do not develop CHRPE.

Most mutations observed around the donor splice site, that cause aberrant splicing, occur at positions -1, +1 or +2 of the intron [19]. There are few reports of mutations altering position +5 [12,21,22]. However, the substitution of the G residue at position +5, as well as at position +1, would be expected to produce a significant reduction of the base pairing stability between the 5' splice site and its complementary region of the U1 snRNA [29,30]. U1 snRNA binding is a crucial step for the correct folding of pre-mRNA prior to cleavage and ligation at the spliceosome.

Moisio *et al *(2002) [12], also report the IVS4+5G>C mutation to cause skipping of exon 4 of the *APC *at the mRNA level and resulting in AFAP. Based on the data presented here and in earlier studies [12,22] we conclude that the IVS9+5G>A mutation is responsible for this patient's AFAP phenotype and that the +5G of the donor site is a highly conserved and functionally important nucleotide.

### Large genomic rearrangements of the *APC*

In this FAP family the patient's phenotype is classic FAP, in agreement with other studies [8-10] supporting that whole gene *APC *deletions produce the classic FAP rather than attenuated FAP. Considering MLPA and FISH results, the size of the identified deletion has a length of about 250 Kbp.

The presence of CHRPE in this patient is in contradiction to the general observation that patients with mutations before exon 9 do not develop CHRPE. Davies *et al *(1995) [31] have also reported a patient with CHRPE and a mutation affecting splicing at the donor splice site of intron 4. Wallis *et al *(1994) [32] state that the presence of CHRPE depends on the size of the truncated APC protein. This leaves a possibility of a fusion transcript as the result of our patient's deletion to be clarified by further experiments such as possibly 3' Rapid Amplification of cDNA Ends (RACE).

Having analysed 25 FAP families in the past 3 years, one family (4%) carrying a large deletion at the *APC *locus was identified. This supports the notion that large genomic events are not rare in *APC *but more data is needed in order to calculate the percentage among Greek patients. Recently, the notion that rearrangements are not so rare for the *APC *but may account for more than 10% of its mutation spectrum, is supported by studies in "*APC *negative" FAP patients. Su *et al *(2000) [6], report a percentage of 13% and Cao *et al *(2001) [16] report 9% of FAP patients carrying large rearrangements. It is possible that the true percentage of these mutations is underestimated due to the limitations of conventional mutation scanning used until now.

### Frameshift and nonsense *APC *mutations

Three small deletions, one small insertion and a nonsense mutation were identified in this study, supporting the fact that most *APC *mutations cause frameshift and usually delete than insert nucleotides. The identified mutations are predicted to produce truncated protein products introducing STOP codons: 1577insT at codon 535, C1690T at codon 564, 1973delAG at codon 672 while 3180delAAAA and 3212delA at codon 1125.

### Genetic testing for adenomatous polyposis

In the past 3 years we have screened 25 families with adenomatous polyposis [4,23]. To minimize time, cost and effort, we use a simple algorithm to perform genetic test for FAP patients. First, we carefully examine the patient's phenotype in order to classify it as classic FAP or AFAP.

For classic FAP we start dHPLC screening of the *APC *from the MCR (Mutation Cluster Region), followed by screening of the remaining 5' region of exon 15. Then we screen the remaining exons with the following order: exons 11 to 14, exons 1 to 10A and finally the remaining exon 15. MLPA is performed in order to identify possible gross genomic alterations affecting the *APC *gene. For AFAP patients, screening follows the order: exons 1- 10A, 3' region of exon 15, exons 11 – 14, 5' region and MCR of exon 15. If no mutation is detected we finally perform the MLPA test.

Even following the above algorithm there is a small number of FAP patients in whom no pathogenic mutation can be detected in the *APC *gene. These cases include patients carrying unclassified variants of these genes such as I1307K, E1317Q and D1822V in the *APC *gene. Large epidemiologic studies and functional studies with transgenic animals are needed to characterize these mutations. Also, prior to examining these cases for promoter mutations or expression defects, reviewing the patient and family history is strongly recommended in order to investigate the possibility that other genes are involved. Recently, numerous studies point out the need for screening the *MYH *gene for biallelic germline mutations in "*APC *negative" FAP patients [33-35]. In these patients, polyposis arises from multiple somatic mutations that accumulate in the *APC *gene due to the biallelic inactivation of the *MYH*.

## Conclusion

In conclusion, the combination of the above methods; PCR, dHPLC, RT-PCR, sequencing and MLPA is a good tool for successful genetic testing of FAP patients. It allows the identification of common but also uncommon or rare mutations of the *APC *gene. Splice site defects or gross genomic alterations may have been underestimated by the older testing methods and require cDNA screening and gene rearrangement testing.

## Competing interests

The author(s) declare that they have no competing interests.

## Authors' contributions

MM carried out the mutation screening, identification and interpretation of the results and preparation of the manuscript and figures, AA contributed in the mutation screening, the preparation of the manuscript and set up the MLPA, HD performed the FISH experiments and their interpretation, VV performed the karyotyping, AP performed the funduscopic examination, AK, KP, ID, JT, GF and NA provided the patient material, diagnosis and managment and GN conceived of the study, and participated in its design and coordination.

All authors read and approved the final manuscript.

## Pre-publication history

The pre-publication history for this paper can be accessed here:
